# Idiopathic Pulmonary Vein Thrombus Extending into Left Atrium: A Case Report and Review of the Literature

**DOI:** 10.1155/2016/3528393

**Published:** 2016-05-05

**Authors:** Muhammad Asim Rana, Nicholas Tilbury, Yashwant Kumar, Habib Ahmad, Kamal Naser, Ahmed F. Mady, Awani Patel

**Affiliations:** ^1^King's Mill Hospital, Sherwood Forest Hospitals NHS Foundation Trust, Nottinghamshire NG17 4JL, UK; ^2^Department of Intensive Care Medicine, King Saud Medical City, Riyadh 11373, Saudi Arabia

## Abstract

Pulmonary vein thrombosis (PVT) is rather an uncommon condition which presents nonspecifically and is usually associated with lung malignancy and major pulmonary surgery. Rarely could no cause be found. It causes increased pulmonary venous pressure leading to pulmonary arterial vasoconstriction and subsequent pulmonary arterial hypertension and subsequently can cause cor pulmonale if not addressed in timely fashion. Other associated complications like peripheral embolization and stroke have also been reported. This case emphasizes the importance of maintaining high index of clinical suspicion especially when CT pulmonary angiogram is negative for pulmonary embolism.

## 1. Case Report

**Figure 1:**
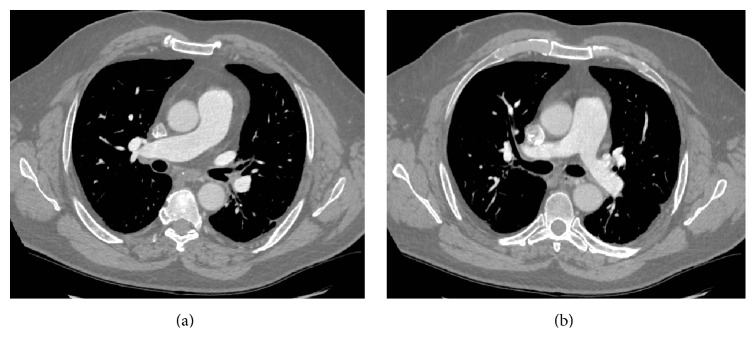
Contrast-enhanced CT pulmonary angiogram showing normal opacification of right and left pulmonary arteries. No evidence of pulmonary embolism.

**Figure 2:**
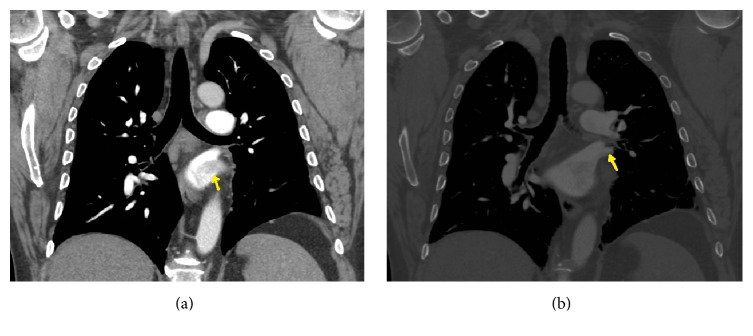
Thrombus (yellow arrow) extending into left atrium.

**Figure 3:**
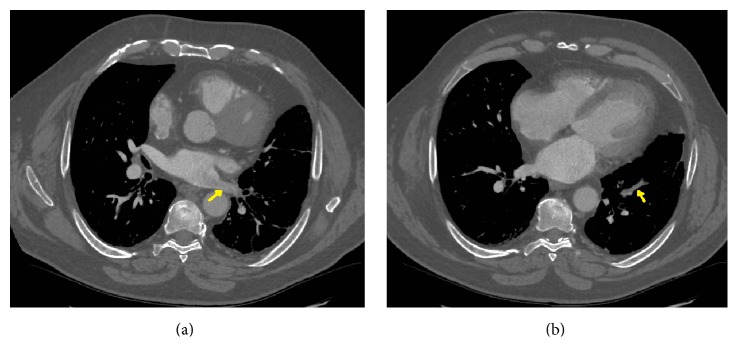
Thrombus in pulmonary vein (yellow arrow) seen in distal tributary as well.

A 63-year-old male patient with no previous medical history and not on any regular medication presented to the emergency department following a one-week history of sudden-onset central chest pain. The pain was pleuritic in nature with associated dyspnoea and palpitations. Physical examination was unremarkable apart from a mild tachycardia of 107 BPM. ECG confirmed sinus rhythm. Baseline blood tests were unremarkable apart from elevated d-dimers of 1800 ng/mL and a chest X-ray showed no acute changes. A working diagnosis of pulmonary embolism was made and a contrast-enhanced CT pulmonary angiogram (CTPA) was performed. The scan showed neither any pulmonary embolism nor any other lung pathology like neoplasia or interstitial lung disease (Figures 1(a) and 1(b)) but did show evidence of a thrombus in the pulmonary vein, extending into the left atrium (Figures 2(a), 2(b), 3(a), and 3(b)). Transthoracic and transoesophageal echocardiograms subsequently confirmed the diagnosis of pulmonary vein thrombosis (PVT). Other echocardiogram findings were normal right atrium with pressure of 0–5 mm Hg, normal right ventricle with good contractility, and pressure of 25–30 mm Hg. Left atrial dimensions were 3.3 cm and systolic pressure was 14.9 mm Hg. Pulmonary artery systolic pressure was 28 mm Hg.

This unusual incidental finding prompted investigation into underlying predisposing factors including thrombophilia screen, antinuclear antibodies, and tumour markers (alpha fetoprotein, beta 2 microglobulin, CA 19-9, and PSA), all of which were normal. No cause of the PVT was found.

Advice was sought from both cardiothoracic surgery and haematology specialists and the most appropriate treatment for this patient was deemed to be oral anticoagulation. A repeat CT scan after 6 months showed complete resolution of the thrombus.

## 2. Discussion

Pulmonary vein thrombosis (PVT) is an uncommon condition that is in most cases associated with major pulmonary surgery and lung malignancies. It has also been seen in cases of trauma, arteriovenous malformations, mitral stenosis, and atrial myxoma and, on occasion, no cause has been found [1].

Presentation of PVT is usually nonspecific, making clinical diagnosis a challenge. Patients may develop shortness of breath, pleuritic chest pain, cough, or haemoptysis and as a result it is not uncommon for it to be misdiagnosed as pulmonary embolism [1]. Most commonly PVT manifests in one of two forms, either an acute pulmonary infarction or a more prolonged course resulting in intermittent pulmonary oedema or pulmonary fibrosis [2].

The pathophysiology of the condition involves an increase in pulmonary venous pressure, which then leads to a compensatory vasoconstriction of the pulmonary arterial vasculature. The subsequent pulmonary arterial hypertension then leads to right ventricular dilatation and, if unchecked, cor pulmonale [3].

There are a number of documented complications associated with PVT. Acute complications include peripheral embolization leading to transient ischaemic attacks or stroke [4, 5]. Secondary infection of the affected lung segment can also occur. If the PVT goes undetected for a prolonged period of time, the patient may develop pulmonary gangrene or, as already mentioned, chronic lung conditions such as pulmonary fibrosis.

There is no gold standard for diagnosis of PVT and usually a combination of diagnostic modalities is required [6, 7]. Chest radiograph may show areas of infarction as patchy opacities but is nonspecific. CT pulmonary angiography is often the initial means of discovering PVT as the mode of presentation usually mimics pulmonary embolism. This is how the diagnosis came to light in this case. Echocardiogram can also be useful, with transoesophageal echocardiogram (TOE) being more sensitive than transthoracic echocardiogram [5] due to the relative close proximity of the pulmonary veins to the distal oesophagus. MR imaging is helpful when differentiating between a tumour and bland thrombus [8].

Our patient presented subacutely with chest pain and dyspnoea with no known pulmonary disease. A CTPA was done to rule out possibility of PE, which showed evidence of PVT extending into the left atrium. In our case we observed the entire length of thrombus starting from pulmonary vein and finishing in the left atrium. The thrombus remained haemodynamically silent and in the absence of embolic phenomenon may have gone unnoticed. The results of the thrombophilia screen and tumour markers were also normal. The primary cause of PVT in this case remains unclear [7, 8] and is keeping with previous evidence base.

Management of PVT depends, to a certain degree, on the underlying cause [2, 9] though, in the absence of bleeding, anticoagulation is usually required. Often, antibiotics are also necessary due to the high risk of secondary infection of any infarcted tissue [10]. When the condition is significantly advanced or the patient deteriorates clinically, lobectomy or even pneumonectomy can be necessary [4, 11]. In our case anticoagulation was achieved with low-molecular weight heparin initially and with warfarin later on. No sufficient data is available on long term management of cases of PVT especially in idiopathic cases treated with anticoagulation.

Pulmonary vein thrombosis is an underrecognized condition/diagnosis with potentially major complications which are preventable (as highlighted in our case) if treated early and should feature in differential diagnosis of pulmonary embolism especially if CTPA is negative.
